# Contribution of on-road transportation to PM_2.5_

**DOI:** 10.1038/s41598-021-00862-x

**Published:** 2021-10-29

**Authors:** Chao Li, Shunsuke Managi

**Affiliations:** Urban Institute and School of Engineering, Kyushu University, 744 Motooka, Nishi-ku, Fukuoka, 819-0395 Japan

**Keywords:** Environmental impact, Environmental impact, Environmental economics

## Abstract

Fine particulate matter (PM_2.5_) mainly originates from combustion emissions. On-road transportation is considered one of the primary sources of PM_2.5_ emission. The relationship between on-road transportation and PM_2.5_ concentration varies temporally and spatially, and the estimation for this variation is important for policymaking. Here, we reveal the quantitative association of PM_2.5_ concentration with on-road transportation by the spatial panel Durbin model and the geographical and temporal weighted regression. We find that 6.17 billion kilometres (km) per km^2^ on-road transportation increase is associated with a 1-μg/m^3^ county-level PM_2.5_ concentration increase in the contiguous United States. On-road transportation marginally contributes to PM_2.5_, only 1.09% on average. Approximately 3605 premature deaths are attributed to PM_2.5_ from on-road transportation in 2010, and about a total of 50,223 premature deaths ascribe to PM_2.5_ taking 6.49% from 2003 to 2016. Our findings shed light on the necessity of the county-level policies considering the temporal and spatial variability of the relationship to further mitigate PM_2.5_ from on-road transportation.

## Introduction

Air pollution, especially fine particulate matter (PM_2.5_), is a severe issue globally that adversely affects human mortality^1–4^, well-being^5,6^, physical health^7,8^, mental health^9^, among others. Several diseases of pulmonary, cardiac, vascular, and neurological systems, including acute lower respiratory illness, cerebrovascular disease, ischaemic heart disease, chronic obstructive pulmonary disease, and lung cancer, are related to PM_2.5_^10^. About 3.3 million premature deaths per year worldwide are attributable to outdoor air pollution, mostly PM_2.5_, and 54,730 of them in the contiguous United States (CONUS, 54,905 in the whole U.S.)^2^. Additionally, other studies demonstrate a more negative situation that roughly 200,000 premature deaths per year are estimated to be caused by PM_2.5_ from combustion emission, and approximately 53,000 deaths are associated with PM_2.5_ from road transportation^11^. Moreover, another report indicates that PM_2.5_ is responsible for 107,000 premature deaths in 2010 in the U.S., and 28% is attributed to transportation^12^. Therefore, PM_2.5_ negatively impacts human health, and road transportation is one of the main reasons for air pollution^11,12^.

On-road transportation is considered one of the primary sources of toxic air pollutants, especially PM_2.5_^13,14^. However, the quantitative association of on-road transportation with measured county-level PM_2.5_ concentration in the CONUS varies temporally and spatially, and the estimation for this variation is important for policymaking. The U.S. Environmental Protection Agency (EPA) claims that transportation is responsible for less than 10% of PM_2.5_ emissions^15^. According to the EPA, the emission sectors of hazardous air pollutants are classified into four types: biogenics, fires, mobile, and stationary^16^. Mobile type of the air pollution emission sectors is generally deemed transportation, which includes aircraft, commercial marine vessels, locomotives, non-road equipment, and on-road vehicles. Previous studies point out that roughly a quarter of PM_2.5_-related mortalities is linked to road transportation in the U.S.^11,12^. Nevertheless, the number of researches on the contribution of the emission sector, on-road vehicles, to PM_2.5_ regarding the U.S. is still limited.

**Figure 1:**
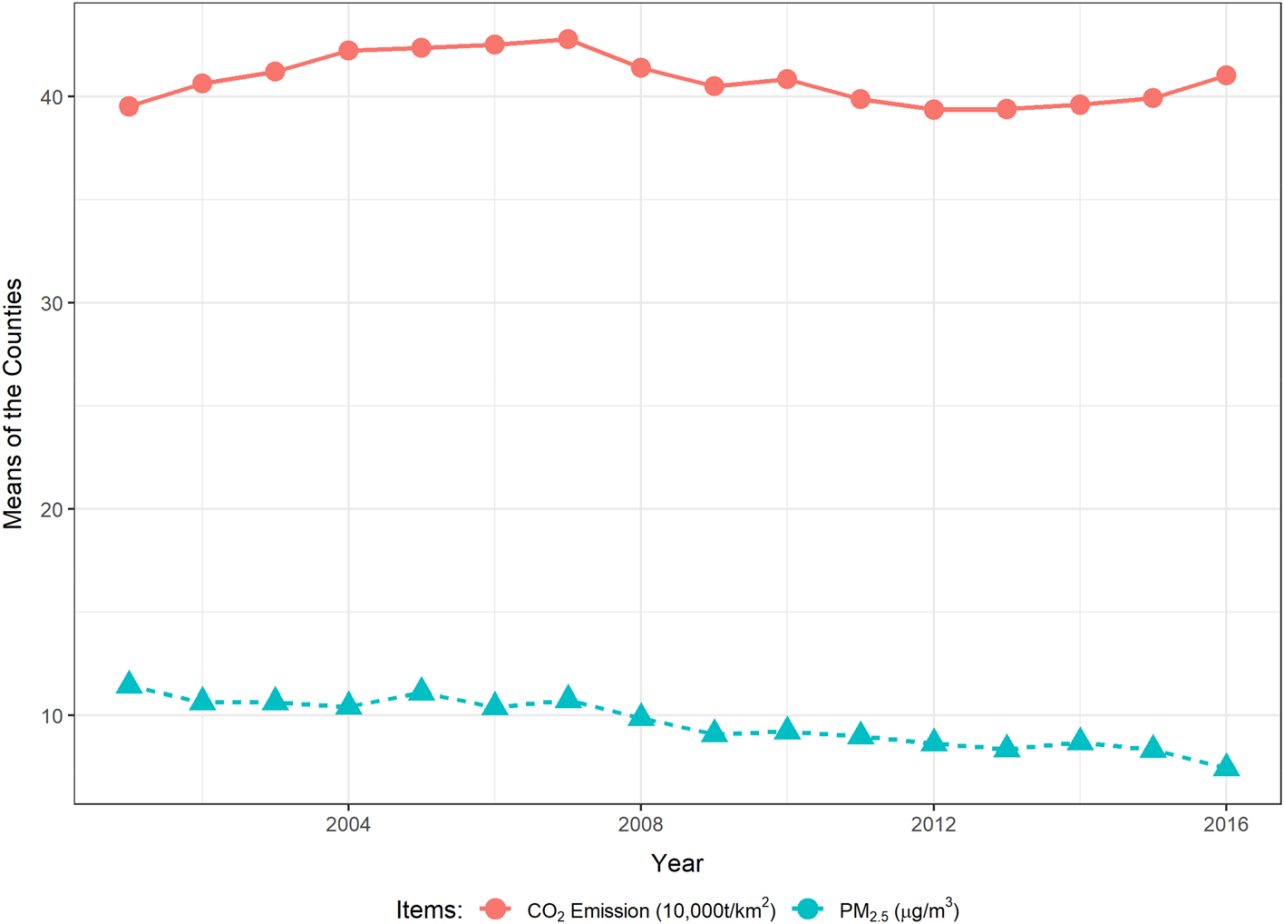
Means of on-road CO_2_ emissions, PM_2.5_ concentrations in the counties of the CONUS (2001–2016) (the means of on-road CO_2_ emissions are the average values of county-level geographically average on-road CO_2_ emissions in the CONUS. The means of PM_2.5_ concentrations are annually average values in each county, based on daily data).

To abate the public health burden, controlling and reducing air pollution emissions from fossil-fuel traffic are practical and beneficial ways^17,18^. The widely implemented strategies include air pollution removal and source control measures. Natural areas, such as forestlands and shrublands, are deemed effective in removing air pollution^19,20^. For instance, 17.4 million tons of air pollution are removed by the trees and forests in the U.S. in 2010^20^. Moreover, the emission source control measures, such as the end-of-pipe control policies and the energy-climate policies, are successful approaches to improve air quality^21,22^. In fact, the average county-level PM_2.5_ concentration in the CONUS continues to decrease obviously from 2001 to 2016, even though counties’ average on-road carbon dioxide (CO_2_) emissions remain relatively stable during this period (as shown in Fig. 1). By the beneficial air pollution control policies, air pollution declined dramatically in the U.S. over the past twenty years, without strongly adversely affecting human daily life^23^. To further mitigate air pollution in future, more detailed information regarding specific emission sectors, e.g., on-road transportation, is desired^12^. Determinations of the primary air pollution sources and estimations of the effectiveness of reduction methods enable policy-makers to formulate reasonable environmental policies without strongly influencing daily human life.

The contribution of transportation to PM_2.5_ concentration varies spatially^12^. Previous studies intensively understand the association of transportation with air pollution in the atmosphere. For example, in Guangdong Province, China, mobile type takes 19.3% responsibility of ambient PM_2.5_^24^; in four main cities in China, Beijing, Shanghai, Guangzhou, and Xi’an, on average 7% PM_2.5_ comes from traffic in January 2013^25^; in a Central European city, Warsaw, Poland, traffic emits about 21% PM_2.5_^26^; and in Hamburg urban area, Germany, traffic contributes with 18% to PM_2.5_ exposure^27^. Furthermore, during different periods, the contributions of on-road transportation to PM_2.5_ concentrations are dissimilar, i.e., the relationship between on-road transportation and PM_2.5_ concentration temporally varies. For instance, because of the advanced end-of-pipe control technologies, air pollution declines in the past 50 years in the U.S., even though the vehicles miles travelled increase^23^. Therefore, both spatial and temporal variation of the relationship between on-road transportation and PM_2.5_ should be considered in the analysis.

The relationship between annually county-level PM_2.5_ concentration and on-road CO_2_ emissions per square kilometre (km^2^) in 3017 counties of the CONUS is investigated based on the panel data from 2001 to 2016. The counties’ geographically average on-road CO_2_ emission each year is employed as the indicator of on-road transportation. As the areas of the counties are noticeably different, the on-road CO_2_ emission per km^2^ is more reasonable than the total value. Because forests and other natural lands reduce air pollution^20^, the land cover data are also controlled. Additionally, the county-level population density, average temperature and relative humidity are taken into account. In the statistical analyses, the fixed effects model (FEM) is performed at first. However, according to previous studies, air pollution significantly impacts distant places rather than only within a region^28^. Spatial dependence tests are applied to the FEM, and the test results show that significantly spatial lag and error correlations exist. The spatial panel Durbin model (SPDM) based on FEM is utilised to estimate the association of air pollution with on-road transportation (detailed reasons for using SPDM in the “Methods” section). Because the SPDM is a typically global model, the estimated parameters are spatially and temporally stationary. The spatial and temporal variability of the relationship between on-road transportation and PM_2.5_ concentration is detected by the geographical and temporal weighted regression (GTWR), based on the spatio-temporally weighted matrix. The results of the GTWR model illustrate that the relationship does vary spatially and temporally. Accordingly, the air pollution mitigation and reduction policies should be adapted to the local situations.

## Results

### Impacts of on-road transportation on PM_2.5_

**Figure 2:**
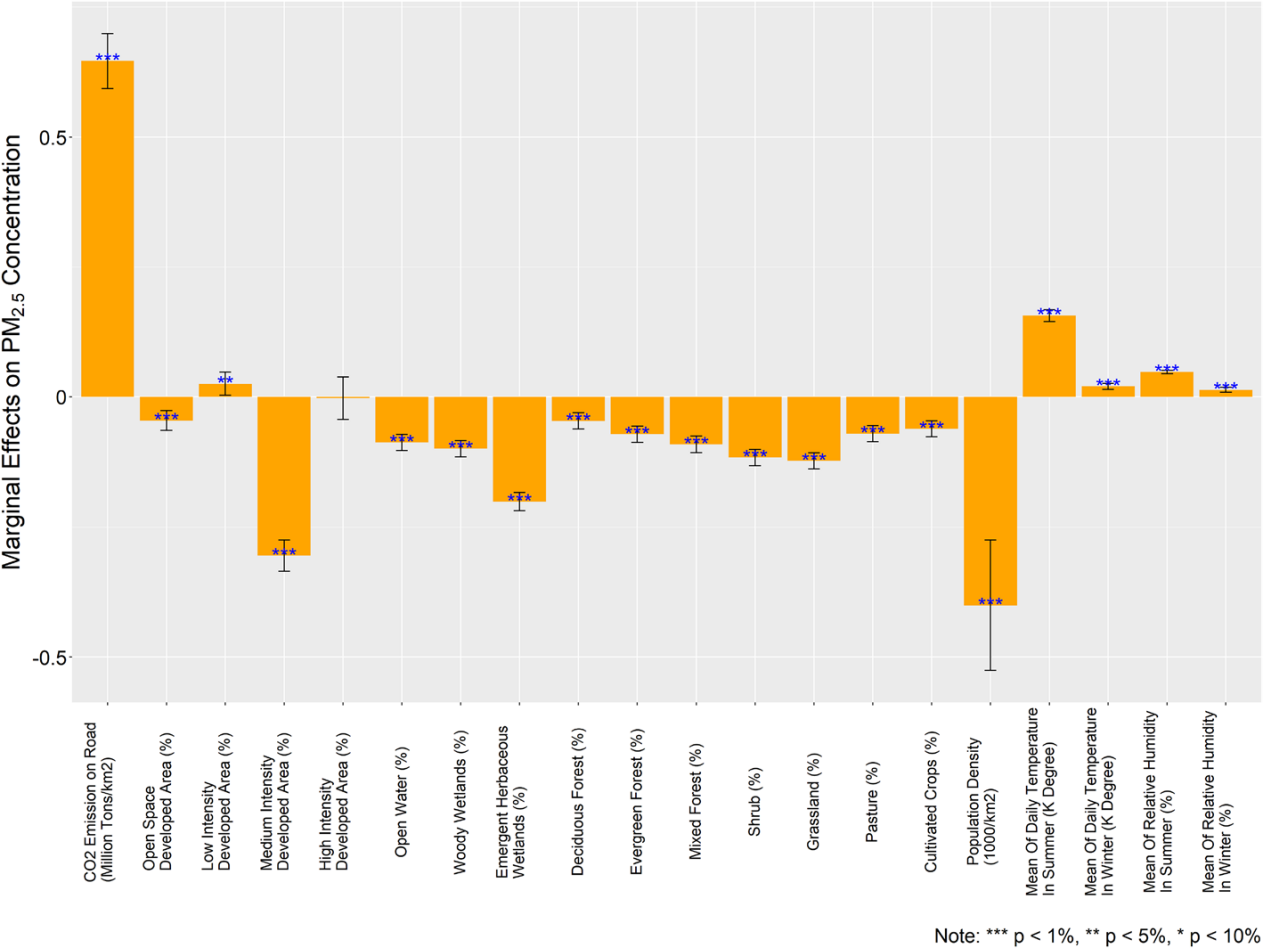
Estimated marginal effects on PM_2.5_ concentration.

Figure 2 illustrates the estimated parameters of the independent variables, adopting the county-level PM_2.5_ concentration as the dependent variable based on the SPDM. The coefficient of the CO_2_ emissions per km^2^ in a county on PM_2.5_ concentration is 0.646 (95% confidence interval: 0.593–0.699). For convenience, CO_2_ emissions are converted into the mileage of typical passage vehicles, where 1 million tons of CO_2_ emissions are roughly equivalent to 3.98 billion km mileage. According to the EPA, the average passenger vehicle emits approximately 404 g of CO_2_ per mile (1 mile = 1.61 km. Hereinafter, mileage unit is based on the average passenger vehicle.). Hence, a 6.17 (95% CI: 5.70–6.71) billion km per km^2^ increase in total mileage in a specific county is associated with a 1-μg/m^3^ increase in the county’s annually average PM_2.5_ concentration (Detailed calculation process is shown in the “Methods” section, Contribution of On-Road Transportation to PM_2.5_ part).

The coefficients of the other control variables in the SPDM are also meaningful. The land cover pattern, population density, and weather conditions are included in the analyses. The percentages of low- and high-intensity developed areas in the counties are positively associated with the air pollution concentrations. In contrast, the percentages of medium-intensity developed areas and developed open space areas are negatively related to the PM_2.5_ concentration. A 1% increase in open space developed area is related to a 0.045-μg/m^3^ increase in the PM_2.5_ concentration and a 1% increase in medium-intensity developed area is associated with a 0.030-μg/m^3^ increase in the PM_2.5_ concentration. In contrast, a 1% increase in low-intensity developed area is correlated with a 0.026-μg/m^3^ decrease in the PM_2.5_ concentration. Accordingly, maintaining a rational development intensity is a significantly more effective approach to reduce air pollution from on-road transportation. In other words, an urban development intensity that is either too high or too low is related to more air pollution, especially PM_2.5_. Emergent herbaceous wetlands, shrublands, and grasslands are negatively associated with PM_2.5_ concentration, but deciduous forestlands are positively correlated with PM_2.5_. High temperature and high relative humidity are associated with more PM_2.5_ concentration in the air.

### Geographical and temporal variations of the impacts

**Figure 3:**
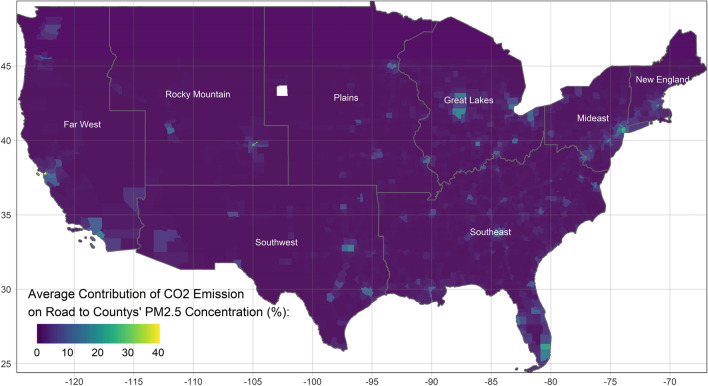
Average contribution of CO_2_ emissions on road to counties’ PM_2.5_ concentration from 2003 to 2016 (this figure is created by R 4.0.4, https://cran.r-project.org/bin/windows/base/old/4.0.4/).

To illustrate the spatial and temporal variability of the coefficients of on-road CO_2_ emissions on the county-level PM_2.5_ concentration, the GTWR is performed. The R^2^ value of the GTWR is higher than that of the global model, which is 0.808. The average impacts of on-road CO_2_ emissions on the PM_2.5_ concentration vary, spatially from east to west, statistically from 0.197 to 0.328 (illustrated in Supplementary Material Fig. S1). Higher values of the coefficients of on-road CO_2_ emissions indicate that on-road transportation exerts greater impacts on air pollution. For example, a unit of on-road transportation increase in New England or the Mideast is associated with a smaller increase in the concentration of PM_2.5_ than in the other regions. In other words, the other air pollution emission sources, except on-road transportation, matter more. Figure 3 shows the average contribution of on-road transportation to air pollution, statistically ranging from 0.002 to 37.765%. Because on-road transportation varies in each county, the spatial distribution of the contribution of on-road transportation to air pollution differs from the spatial distribution of the average effects of on-road transportation. The average contributions of on-road transportation in metropolitan areas, such as New York, San Francisco, Denver, and St. Louis, exceed 20%. In contrast, in rural areas, the average contributions are lower than 1%. In addition, the average contribution of on-road transportation to air pollution reaches only 1.09% (95% CI: 1.06–1.11%), which is lower than the value estimated by the EPA^15^.

Furthermore, the impacts of on-road transportation on air pollution temporally vary based on the results of the GTWR. According to Table 1, the average annual on-road CO_2_ emissions in the counties remain relatively stable, which indicates that on-road transportation did not change dramatically. However, the average contribution of on-road transportation to air pollution reached the lowest point in 2008, only 0.60% on average. These results demonstrate that the structure of air pollution sources might change in 2008 due to the financial crisis (Figure S2 shows the spatial distribution of the annual contribution of on-road transportation to county-level PM_2.5_).

**Table 1:** Mean of on-road CO_2_ emission, coefficients of on-road CO_2_ emission on air pollution, and contribution of on-road transportation.

Year	N	On-road CO_2_ emission		Coefficient		Contribution	
		Mean	SD	Mean	SD	Mean	SD
2003	3107	0.412	1.123	0.440	0.039	1.386	3.100
2004	3107	0.422	1.159	0.317	0.033	1.029	2.342
2005	3107	0.424	1.167	0.302	0.036	0.922	2.129
2006	3107	0.425	1.180	0.277	0.032	0.929	2.152
2007	3107	0.428	1.188	0.201	0.027	0.655	1.552
2008	3107	0.414	1.146	0.181	0.034	0.601	1.394
2009	3107	0.405	1.116	0.196	0.026	0.751	1.825
2010	3107	0.408	1.107	0.258	0.030	0.995	2.408
2011	3107	0.399	1.051	0.328	0.037	1.221	2.697
2012	3107	0.394	1.017	0.335	0.031	1.357	3.093
2013	3107	0.394	1.003	0.356	0.036	1.435	3.122
2014	3107	0.396	1.005	0.316	0.036	1.230	2.707
2015	3107	0.399	1.026	0.340	0.032	1.400	3.119
2016	3107	0.410	1.095	0.268	0.019	1.294	3.121

The unit of on-road CO_2_ emission is 10^4^ t/km^2^, and the contribution of on-road CO_2_ emission on air pollution is the percentage.

The coefficients in the table indicate the relationship between on-road CO_2_ emission and annually county-level concentration, according to the estimation of the GTWR. Because the local model, GTWR, provide temporally and spatially non-stationary coefficients, the coefficients should be different county by county, year by year. We calculate the means and standard deviations of coefficients in each county annually.

The contributions are the means and standard deviations of the contributions in each county annually, based on the calculation of Eq. (9).

### PM_2.5_-related premature deaths due to on-road transportation

**Figure 4:**
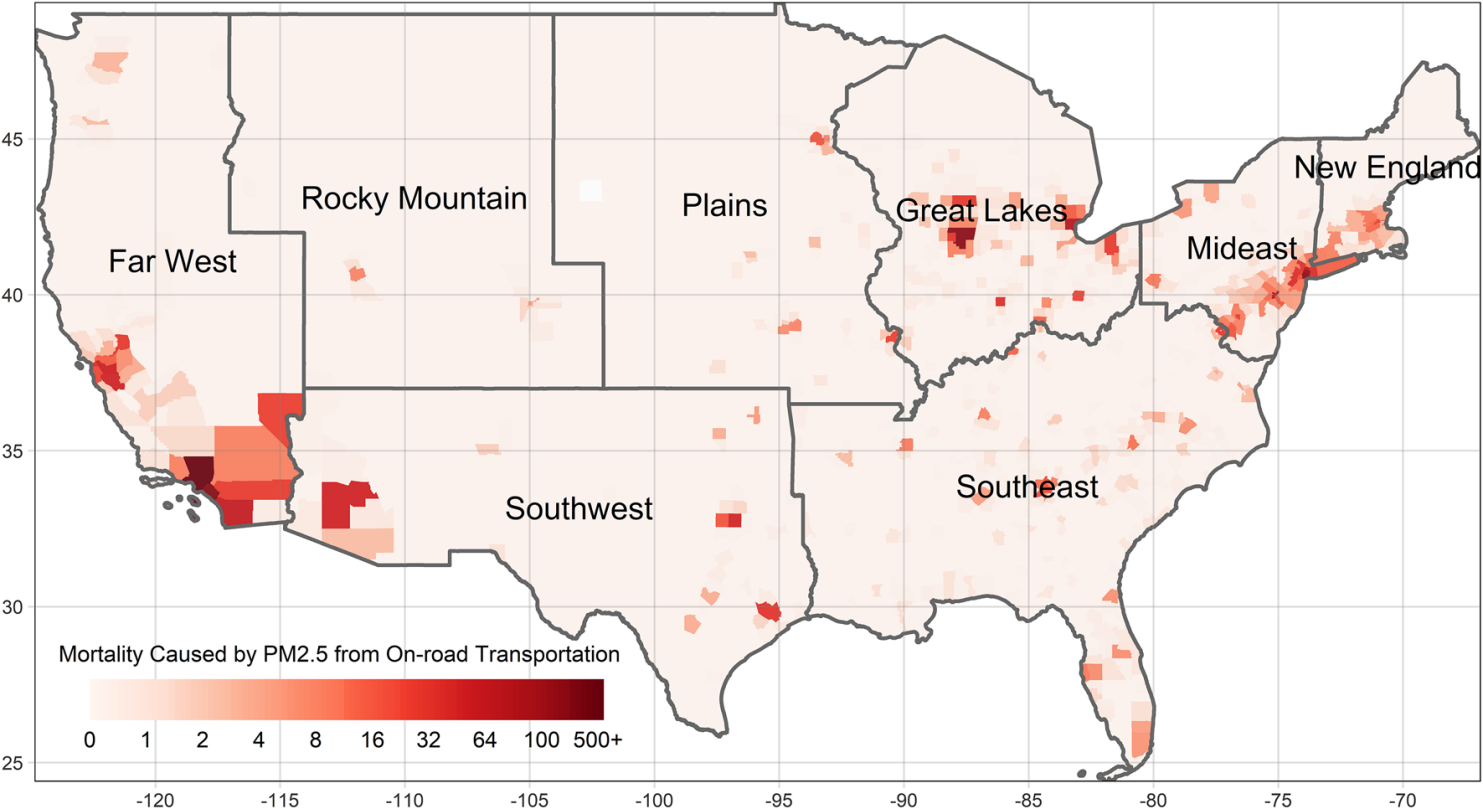
Mortality caused by PM2.5 from on-road transportation in 2010 (this figure is created by R 4.0.4, https://cran.r-project.org/bin/windows/base/old/4.0.4/).

Figure 4 illustrates the spatial distribution of the estimated mortality caused by PM_2.5_ stemming from on-road transportation in each county. The numbers of premature mortalities in each county range from 0 to 1750 in 2010. Moreover, the conditions are severe in metropolitan areas. Approximately 3605 deaths are caused by air pollution originating from on-road transportation, which accounted for 6.59% of the total PM_2.5_-related mortalities (54,730 deaths) in 2010. From 2003 to 2016, approximately 50,223 premature deaths are caused by air pollution stemming from on-road transportation, at 6.49% of the estimated total premature deaths caused by PM_2.5_ (Estimations of each year is listed in Table 2, calculation process can be found in the “Methods” section, Impacts of PM_2.5_ from On-road Transportation on Premature Mortality part).

**Table 2:** Estimated PM_2.5_-related premature deaths due to all sources and on-road transportation.

Year	Premature deaths due to PM_2.5_	Premature deaths due to PM_2.5_ from on-road transportation	Percentage
2003	56,892	4447	7.82
2004	56,471	3423	6.06
2005	56,790	3150	5.55
2006	56,106	3217	5.73
2007	56,319	2280	4.05
2008	55,722	2125	3.81
2009	54,926	2693	4.90
2010	54,730	3605	6.59
2011	54,746	3958	7.23
2012	54,423	4361	8.01
2013	54,302	4427	8.15
2014	54,642	3818	6.99
2015	54,241	4446	8.20
2016	53,732	4274	7.96
Total	774,042	50,223	6.49

Premature deaths due to PM_2.5_ in each county are estimated using Eq. (12), premature deaths due to PM_2.5_ from on-road transportation in each county are calculated using Eq. (11), and the percentage is assessed as Eq. (13).

## Discussion

On-road transportation is associated with PM_2.5_ concentrations in the counties, but it is not the dominant source of air pollution in the U.S. Furthermore, the negative impact of on-road transportation on air quality is not so strong as that reported by the government^15^. An increase in vehicle usage of approximately 6.17 billion km per km^2^ is related to only a 1-μg/m^3^ increase in PM_2.5_ concentration in the counties of the CONUS, and on-road transportation only contributes 1.09% (95% CI: 1.06–1.11%) to PM_2.5_ on average. Additionally, only 6.49% of PM_2.5_-related premature mortalities are attributed to on-road transportation in the CONUS from 2003 to 2016.

### Impacts of on-road transportation on PM_2.5_

On-road transportation is widely considered one of the major sources of air pollution, proved by the previous studies^25,29^. This study finds that on-road transportation CO_2_ emission is positively associated with the PM_2.5_ concentration. However, in terms of the contribution of on-road transportation to PM_2.5_, it is lower. On average, 1.09% (95% CI: 1.06–1.11%) of the PM_2.5_ concentration is attributable to on-road transportation, in line with the value lower than 10% attributed to all transportation reported by the EPA^15^, in a way. Furthermore, our analysis reveals that an approximately 6.17 billion km per km^2^ reduction of on-road transportation is associated with a 1-μg/m^3^ reduction in PM_2.5_. Therefore, the outcomes of strategies striving to reduce on-road vehicle usage should be marginal. Moreover, only 6.49% of the air pollution-related mortality, namely, 50,223 deaths, is associated with air pollution originating from on-road transportation from 2003 to 2016. Most of these deaths occur in metropolitan areas due to the high contribution of on-road transportation to air pollution and high population density.

### Temporal variation of the impacts on PM_2.5_

Additionally, the composition of the air pollution sources is seemingly related to the economic situation. During the 2008 financial crisis, the contribution of on-road transportation to air pollution was the lowest in the 14 years. The contribution of on-road transportation continued to decrease from 2003 to 2008 and increased after 2008. This contribution decrease might be caused by the change in the composition of air pollution sources^23^. The abovementioned change suggests that the enhanced effects of other air pollution sources might affect more during this economic recession period. Furthermore, the EPA published the Final Rule for Control of Hazardous Air Pollutants from Mobile Sources in 2007 to further reduce air pollutants from mobile sources^22^. However, on-road transportation remained stable during this period. Therefore, the variation in other air pollution emission sectors might be the main reason for the temporal variation of the contributions of on-road transportation to air pollution. In a way, the investments in the reduction of other pollution sources should be more effective.

### Spatial variation of the impacts on PM_2.5_

Natural environments, such as vegetated areas, are critical ways to control and prevent air pollution diffusion^19,20^. Air pollutants, especially PM_2.5_, mainly originate from precursor emissions, such as nitrogen oxides, sulfur oxides, and ammonia, through atmospheric chemical reactions^30,31^. According to this study, the contribution of on-road transportation to air pollution is higher in metropolitan areas than that in other areas, consistent with previous studies^12^. Thus, air pollution restrictions on on-road transportation should be strict in urban areas, while the policies could be loose in rural areas. The traffic volume in metropolitan areas is much higher than that in other areas, while less natural land cover occurs. A key reason might be that the natural purification ability is exceeded in those areas. In addition, widely implemented clean fuels and stringent vehicle engine regulations are the critical factors in precursor pollutants emissions reduction^22^. Specifically, the same amount of combustion produces less toxic and lower pollutants emissions, eventually reducing air pollution without dramatically influencing daily human life. However, the reduction mainly attributed to other pollutant sources is also reasonable because the air pollutants discharged by polluting industries and factories are ignored due to a lack of data. Owing to stringent regulations and economic globalisation, the air pollutants originating from polluting industries and factories might notably be reduced^23^. The increase in air pollution stemming from on-road transportation is masked by reducing emissions from industries and factories. Therefore, we might underestimate the effects of on-road transportation on air pollution, although we adopted spatial models to reduce this possibility.

### Future research

Overlooking the air pollutant emissions of polluting industries and factories is a significant limitation. In addition, the considered air pollution data are county-level records, which might cause ecological fallacy. Finally, the chemical mechanisms of the impacts of on-road transportation on air pollution are not considered, so the results cannot be greatly and further explained. In future studies, the mechanism between on-road transportation and the ground-level air pollution concentration should be added to the analyses. More detailed remote sensing data with high temporal and spatial resolution should be generated and applied to monitor other emission sectors. Furthermore, more information about vehicles, such as vehicle type, fuel types, quantity, the technology of engines, among others, should be considered to examine the causes of temporal and spatial variation of the contribution of on-road transportation to PM_2.5_ in future. Additionally, the relationships between land cover patterns and air pollution require further examination, providing informative suggestions for urban planning strategies.

## Conclusion

Our study illustrates the quantitative relationship between on-road transportation and PM_2.5_ concentration, taking temporal and spatial variability into account. On-road transportation contributes to PM_2.5_, but the impact of transportation is marginal, which is only 1.09% on average. We also find that a 6.17 billion km per km^2^ on-road transportation increase is associated with a 1-μg/m^3^ county-level PM_2.5_ concentration increase. Moreover, from 2003 to 2016, about a total of 50,223 premature deaths are attributable to PM_2.5_ stemming from on-road transportation, which takes 6.49% of estimated PM_2.5_-related premature deaths. Most of them are located in metropolitan areas. In addition, the relationship does vary temporally, and economic status and environmental policies might be the reasons for this variation. County-level strategies aiming at reducing PM_2.5_ from on-road transportation should account for the temporal and spatial variability of the relationship.

## Materials and methods

### Materials

#### County-level PM_2.5_ concentrations

The county-level fine particulate matter (PM_2.5_) concentration in the contiguous United States (CONUS) is applied as the dependent variable in all regression models. The data are obtained from the Centers for Disease Control and Prevention, as estimated with the downscaler model by the Environmental Protection Agency (EPA) (more details about this dataset: https://www.cdc.gov/nceh/tracking/topics/AirQuality.htm). The data include the estimated daily PM_2.5_ concentration in each county from 2001 to 2016. We calculate the mean annual PM_2.5_ concentration in each county, and the unit is microgram per cubic metre (μg/m^3^). Because the on-road carbon dioxide (CO_2_) emission data, land cover data, and population density data are annual, lacking daily variation, the PM_2.5_ concentration is converted into an annual average value.

#### On-road county-level CO_2_ emissions

The average annual CO_2_ emissions per square kilometre (km^2^) in each county are adopted as the critical independent variable. First, we obtain the annual on-road CO_2_ emissions at a 1-km resolution from 2001 to 2016 from the National Aeronautics and Space Administration (more details about this dataset: https://daac.ornl.gov/cgi-bin/dsviewer.pl?ds_id=1735)^32^. The county boundaries in the CONUS are acquired from the U.S. Census Bureau, created in 2016. The on-road county-level CO_2_ emissions are expressed in units of million tons per km^2^. Because the areas of the different counties vary dramatically, the total CO_2_ emissions in each county cannot be compared. In this case, the total CO_2_ emissions in each county are not strongly associated with the air pollution concentration, but the CO_2_ emissions per km^2^ in each county are. Additionally, CO_2_ emissions are predicted based on data retrieved from the Federal Highway Administration’s Highway Performance Monitoring System expressed in roadway-level vehicle miles^33,34^. In other words, the CO_2_ emissions in the counties indicate vehicle usage^35^.

#### Land cover data

We exploit in 2001, 2004, 2006, 2008, 2011, 2013, and 2016 land cover datasets^36,37^ with a 30-m resolution in the CONUS acquired from the National Land Cover Dataset (NLCD: https://www.mrlc.gov/data/nlcd-land-cover-conus-all-years). The NLCD includes 16 land types: open water, perennial ice, developed open spaces, low-intensity developed areas, medium-intensity developed areas, high-intensity developed areas, barren areas, deciduous forestlands, evergreen forestlands, mixed forestlands, shrublands, grasslands, pastures, cultivated croplands, woody wetlands, and emergent herbaceous wetlands. The land cover variables are the percentages of each land type in every county. We linearly interpolate the data in missing years.

#### Other control variables

The population density, mean daily summer and winter temperatures, and means of relative humidity in summer and winter in every county are also controlled. The population density data are the raster data provided by the University of Southampton from 2001 to 2016 with a 100-m resolution and estimated via unconstrained top-down methods (details about WorldPop: https://www.worldpop.org/project/categories?id=18). The unit of the population density is thousand people per km^2^^38^. Meteorological variables are also the grid data, such as the temperature and relative humidity in winter and summer, from 2001 to 2016 with a 4-km grid size in each county (more details about meteorological variables: http://www.climatologylab.org/gridmet.html, a statistical data summary is provided in Table S1, and the data sources are listed in the Table S2).

### Methods

To explore the role of spatial heterogeneity in the relationship between vehicle usage and county-level PM_2.5_ concentration, five regression models are employed in our research, including the fixed effects model (FEM), spatial panel autoregressive model (SPAM), spatial panel error model (SPEM), spatial panel Durbin model (SPDM)^39^, and geographical and temporal weighted regression (GTWR)^40^.

#### FEM

The FEM is the basic regression model among all five models. The FEM is preferred in this research, according to a series of statistical tests. Based on the F test for individual effects, the results are significant, adopting PM_2.5_ concentration as the dependent variable, which indicates that the FEM is better than the ordinary least square (OLS) method. According to the Breusch–Pagan Lagrange Multiplier Test for balanced panel data, the significant results indicate that the random effects model (REM) is also better than the OLS method. Finally, in the view of the Hausman test, the significant results demonstrate that the FEM performs better than the REM. The FEM can be expressed as:

1 $$AP_{it} = {\varvec{\beta}}_{1} {\varvec{X}}_{it}^{\prime } + \alpha_{i} + { }\mu_{it}$$

where $AP_{it}$ denotes the mean PM_2.5_ concentration (μg/m^3^) in county $i$ during year $t$, ${\varvec{X}}_{it}$ denotes a vector of the independent variables in county $i$ during year $t$, $\alpha_{i}$ denotes the unobserved time-invariant individual effect, $\mu_{it}$ denotes the idiosyncratic error, and ${\varvec{\beta}}_{1}$ is a vector of the parameters to be estimated. The vector, ***X***, of independent variables includes on-road CO_2_ emission (million tons/km^2^), open space developed area (%), low intensity developed areas (%), medium intensity developed areas (%), high intensity developed areas (%), open water (%), woody wetlands (%), emergent herbaceous wetlands (%), deciduous forest (%), evergreen forest (%), mixed forest (%), shrub (%), grassland (%), pasture (%), cultivated crops (%), population density (thousand capita/km^2^), mean of daily temperature in summer (K°), mean of daily temperature in winter (K°), mean of relative humidity in summer (%), and mean of relative humidity in winter (%) (the data statistic summary is shown in Table S1).

The FEM assumes that the observations and error terms are independent, similar to the OLS method. However, air pollutants can diffuse in air, resulting in cross-county and even cross-state impacts^28^. In other words, the observations exhibit spatial correlations, which the FEM ignores. Therefore, we also apply four other models, including the SPAM, SPEM, SPDM, and GTWR, to investigate the spatial relationships among the variables.

#### SPAM

The SPAM assumes that a spatial correlation exists between the dependent variable of a given observation and its neighbours^39^. In our research, the average PM_2.5_ concentration in a county is related to the county-level air pollutant concentration in its surrounding counties. As an improvement of the FEM, a spatial autoregressive part is added to the SPAM. According to the locally robust Lagrange Multiplier test to determine spatial lag correlations, the result is significant, adopting PM_2.5_ concentration as the dependent variable. Therefore, the SPAM for analysing our dataset to detect the relationship between on-road transportation and the PM_2.5_ concentration in the counties is reasonable. The SPAM is expressed as follows:

2 $$AP_{it} = \lambda {\varvec{W}}_{i} {\varvec{NEAP}}_{it}^{\prime } + {\varvec{\beta}}_{1} {\varvec{X}}_{it}^{\prime } + \alpha_{i} + \mu_{it}$$

where ${\varvec{W}}_{i}$ is a vector of the spatial weights of the neighbours of county $i$, ${\varvec{NEAP}}_{it}$ is a vector of the county-level PM_2.5_ concentrations (μg/m^3^) in the neighbouring counties of county $i$ during year $t$, and $\lambda$ is the spatial lag parameter to be estimated. The Queen method is employed to calculate the spatial weight vectors of each county. If two counties share only one point, they are deemed contiguous in the Queen method^41^. The spatial weights in vector ${\varvec{W}}_{i}$ is the quotient of 1 and the count of the elements in vector ${\varvec{NEAP}}_{it}$. In the SPAM, the neighbouring counties affect county $i$, but the county $i$ also reversely influences these neighbouring counties. Therefore, the SPAM involves an iterative calculation process to figure out the spatial spillover function^42^. According to the recommendation of the package maker, the number of iterations is generally set to 500 to estimate the regression coefficients correctly^39^.

#### SPEM

The SPEM supposes that there is a spatial dependence in the error term of the FEM^39^. Thus, the error term of the FEM is divided into a spatially related part and a spatially unrelated part. According to the locally robust Lagrange Multiplier test to determine spatial error correlations, the result is significant, adopting PM_2.5_ as the dependent variable. The SPEM is, thus, reasonable for the analysis. The SPEM is expressed as follows:

3 $$AP_{it} = {\varvec{\beta}}_{1} {\varvec{X}}_{it}^{\prime } + a_{i} + \rho {\varvec{W}}_{i} {\varvec{\varepsilon}}_{it}^{\prime } + \nu_{it}$$

where ${\varvec{\varepsilon}}_{it}$ denotes the spatially related error term of county $i$ during year $t$, $\upsilon_{it}$ denotes the spatially unrelated error term of county $i$ during year $t$, and $\rho$ is the spatial correlation parameter to be estimated.

#### SPDM

The SPDM accepts the assumptions of both the SPAM and SPEM. The SPDM is expressed as:

4 $$AP_{it} = \lambda {\varvec{W}}_{i} {\varvec{NEAP}}_{it}^{\prime } + { }{\varvec{\beta}}_{1} {\varvec{X}}_{it}^{\prime } + \alpha_{i} + \rho {\varvec{W}}_{i} {\varvec{\varepsilon}}_{it}^{\prime } + \nu_{it}$$

where $\lambda$ and $\rho$ are the spatial correlation parameters to be estimated. Additionally, because of the spatial spillover effect, the number of iterations is set to 500. When the results of the locally robust Lagrange Multiplier tests to determine spatial lag and spatial error correlations are significant, the SPDM is considered the best global model.

### GTWR model

To account for the local effects of the independent variables in our research, the GTWR model is also exploited. The GTWR model is extended from the geographically weighted regression (GWR) model based on spatial panel data. The current GWR model generally employs the OLS method as the basic algorithm to estimate local coefficients. However, our dataset contains balanced panel data, so any local effects in time should also be considered. In contrast to the GWR model, the GTWR model assumes that the relationships between the dependent and independent variables are spatially and temporally non-stationary^40,43^. The GTWR model divides the total sample into numerous subsamples according to the spatial context and temporal extent. The critical issue of this division is to decide the subsample size, namely, the bandwidth^40^. In this study, the cross-validation method is applied to minimise the sum of the squared error^44^. In addition, the bandwidth is an adaptive spatio-temporal distance, rather than a fixed spatio-temporal distance, to guarantee that each subsample exhibits the same size. The GTWR model is expressed by:

5 $$AP_{itk} = \beta_{0itk} { } + { }\mathop \sum \limits_{k} {\varvec{\beta}}_{itk} {\varvec{X}}_{itk}^{\prime } + \varepsilon_{i} ,\;i = 1, \ldots ,n$$

where $AP_{itk}$ denotes the annually average PM_2.5_ concentration (μg/m^3^) in county $i$ during year $t$ in the $k$ th subsample, ${\varvec{X}}_{itk}$ denotes a vector of the independent variables in county $i$ during year $t$ in the $k$ th subsample, $\beta_{0itk}$ is the intercept in county $i$ during year $t$ of the $k$ th regression, ${\varvec{\beta}}_{itk}$ denotes a vector of the parameters in $k$ th regression in the county $i$, $n$ denotes the number of observations in the $k$ th regression, and $\varepsilon_{i}$ is the error term.

The coefficients of the GTWR are estimated as follows:

6 $${\varvec{\beta}}_{itk} = \left[ {{\varvec{X}}^{T} {\varvec{W}}_{itk} {\varvec{X}}} \right]^{ - 1} {\varvec{X}}^{T} {\varvec{W}}_{itk} {\varvec{Y}}$$

where ${\varvec{W}}_{itk}$ is the spatio-temporally weighted matrix of county $i$ during year $t$ in the $k$ th regression to estimate the coefficients. ${\varvec{W}}_{itk}$ is based on the spatio-temporal distance among the records^40^. There are 49,712 records in the dataset pertaining to 3,107 counties, from 2001 to 2016, so the size of ${\varvec{W}}_{itk}$ is roughly $2.47{ } \times { }10^{9}$, which unfortunately exceeds the limitation of R. Thus, we have to eliminate the data of two years, namely, 2001 and 2002. The spatio-temporally weighted matrix is calculated as follows:

7 $${\varvec{W}}_{itk} = \left\{ {\begin{array}{*{20}l} {exp\left( { - \frac{{{\varvec{d}}_{it}^{2} }}{{b_{it}^{2} }}} \right),} \hfill & {if\;element\left( {it} \right) \in subsample\;k} \hfill \\ {0,} \hfill & {if\;element\left( {it} \right) \notin subsample\;k} \hfill \\ \end{array} } \right.$$

where ${\varvec{d}}_{it}$ is a vector of the combination of the geographical and temporal distances between county $i$ and its neighbouring counties in the $j$ th subsample, and $b_{it}$ is the largest distance in the vector ${\varvec{d}}_{it}$. $b_{it}$ is determined based on the optimal bandwidth because we use the adaptive bandwidth. The Euclidean distance is determined to obtain the spatio-temporal distance, and the spatio-temporal distance is calculated as follows:

8 $${\varvec{d}}_{it}^{2} = \user2{ }\varphi_{S} {\varvec{dS}}_{it}^{2} + \varphi_{T} {\varvec{dT}}_{it}^{2}$$

where $\varphi_{S}$ is the scale factor of the spatial distance, $\varphi_{S}$ is the scale factor of temporal distance, ${\varvec{dS}}_{it}$ is a vector of the spatial distance between county $i$ and its neighbouring counties in the $j$ th subsample, and ${\varvec{dT}}_{it}$ is a vector of the temporal distance. Moreover, $\varphi_{S}$ and $\varphi_{T}$ can be determined once the bandwidth is selected.

### Models selection

The FEM and SPEM results are summarised in Table S3, and the R^2^ values of the FEM and SPEM are 0.366 and 0.491, respectively. The direct, indirect, and total impacts on the county-level PM_2.5_ concentration are estimated by the SPAM and SPDM, respectively (listed in Tables S4 and S5). The so-called impact here is a term obtained from R package “spdep”^42^, but its real meaning is the coefficient. The direct impacts indicate the direct relationships between the independent and dependent variables of a given county. In other words, direct impacts are the coefficients estimated by Eqs. (2) or (4) without any iteration. The indirect impacts arise from the changes in the ${\varvec{NEAP}}_{it}$, which is the dependent variable of a specific county’s neighbourhoods. For example, New York county’s PM_2.5_ concentration affects Kings county’s, but Kings county’s also influences New York county’s. In mathematics, this spillover would be endless. However, the spatial spillover function is monotonically increasing concave function and converges towards a certain value. The coefficients estimated according to spatial spillover function are called indirect impacts^42^. Simply speaking, the indirect impacts can be deemed that New York county’s on-road transportation causes PM_2.5_, which partially diffuses to Kings county and then come back again. The coefficient of the on-road transportation on the PM_2.5_ spread back is its indirect impact. The total impact is the accumulative value of the direct and indirect impacts. According to the locally robust panel Lagrange Multiplier tests, the spatial correlations of both dependent variable and error term are significant at 0.1% level when adopting the PM_2.5_ concentration as the dependent variable. In this case, the SPDM is the most reasonable global model. In light of the abovementioned statistical tests and findings, the SPDM is deemed the best global model to analyse the relationship between the county-level PM_2.5_ concentration and on-road transportation CO_2_ emissions (R^2^ of each model and statistical tests for models selection are recorded in Tables S6 and S7, respectively). Additionally, to detect the spatial and temporal variability, the GTWR model is also considered. Among all five models, the interpretation of the GTWR is the highest (R^2^ = 0.808, listed in Table S6).

### Contribution of on-road transportation to PM_2.5_

The coefficient of on-road CO_2_ emission is 0.646 (0.593–0.699) based on the SPDM (see Table S5). This coefficient is the total impact, the sum of the direct and indirect impacts. The coefficient means a 1 million tons on-road CO_2_ emission increase per km^2^ in a specific county is related to a 0.646 μg/m^3^ increase in the county’s PM_2.5_ concentration. In other words, 1 μg/m^3^ PM_2.5_ from on-road transportation in a certain county is associated with about 1.55 million tons on-road CO_2_ emission per km^2^. According to the EPA (https://www.epa.gov/greenvehicles/greenhouse-gas-emissions-typical-passenger-vehicle), the average passenger vehicle emits approximately 404 g of CO_2_ per mile (1 mile = 1.61 km). It must be noted that here the conversion factor is on the basis of the passenger vehicle. Thus, 1 million tons on-road CO_2_ emission is equivalent to 3.99-billion-km mileage of the average passenger vehicle. Finally, according to the all numbers listed above, a 6.17 (5.70–6.71) billion km per km^2^ increase in total mileage in a specific county is associated with a 1-μg/m^3^ increase in the county’s annually average PM_2.5_ concentration.

The contribution of on-road transportation to air pollution based on the GTWR model is estimated as follows:

9 $$Con_{it} = \frac{{\beta_{it} \times {\text{CO}}_{2it} }}{{Concentration_{it} }}{ } \times 100{\text{\% }}$$

where $Con_{it}$ denotes the contribution (as a percentage) of on-road transportation to air pollution in county $i$ during year $t$, $\beta_{it}$ is the estimated coefficient of the impact of on-road CO_2_ emissions per km^2^ on air pollution in county $i$ during year $t$ with the GTWR model, ${\text{CO}}_{2it}$ denotes the average on-road CO_2_ emissions per km^2^ during the 14-year study period in county $i$ during year $t$, and $Concentration_{it}$ is the average concentration of air pollutant concentration in county $i$ during year $t$. The average contribution of on-road transportation to annually county-level PM_2.5_ concentration is 1.09% (range from 0% to 60.68%, 95% CI: 1.06–1.11%).

### Impacts of PM_2.5_ from on-road transportation on premature mortality

The results obtained from the GTWR model are applied to estimate the effects of air pollution on PM_2.5_-related mortality. Different from the coefficients estimated by the global models, the GTWR model indicates that the relationship between on-road transportation and PM_2.5_ concentration is temporally and spatially non-stationary. According to the calculations with Eq. (9), the contribution of on-road transportation to air pollution might either exceed 100% or be lower than 0%. Due to the effects of the other control variables and regression residuals, the contributions might exceed 100%. Additionally, a contribution lower than 0% suggests that on-road transportation emissions are a method to reduce air pollution, which is impossible and irrational. To estimate the mortality in the CONUS, we normalise the contribution of on-road transportation from 0 to 100%, as follows:

10 $$C_{it} = \left\{ {\begin{array}{*{20}l} {0,} \hfill & {if\;Con_{it} < 0} \hfill \\ {Con_{i} ,} \hfill & {if\;0 \le Con_{it} \le 100\% } \hfill \\ {100\% ,} \hfill & {if\;Con_{it} > 100\% } \hfill \\ \end{array} } \right.$$

where $C_{i}$ denotes the contribution of on-road transportation to air pollution ranging from 0 to 100% in county $i$ and $Con_{it}$ denotes the contribution (as a percentage) of on-road transportation to air pollution in county $i$ during year $t$ calculated with Eq. (9). It must be noted that all the $Con_{it}$ based on the results of the GTWR are larger than 0 and smaller than 100%. The mortalities caused by air pollution originating from on-road transportation are also estimated as follows:

11 $$MAPOR_{it} = MAP_{it} \times C_{it}$$

where $MAPOR_{it}$ is the number of mortalities caused by air pollution originating from on-road transportation in county $i$ during year $t$, and $MAP_{it}$ is the number of mortalities caused by PM_2.5_ in county $i$ during year $t$. $MAP_{i2010}$ data are extracted from the previous study^2^. The premature deaths $MAP_{it}$ in the CONUS are estimated as follows:

12 $$MAP_{it} = MAP_{it - 1} \times \left[ {1 + CPD \times \left( {AP_{it} - AP_{it - 1} } \right)} \right]$$

where $MAP_{it - 1}$ is the number of mortalities caused by PM_2.5_ in county $i$ during year $t - 1$, $CPD$ is the coefficient between the increase of premature deaths and the increase of PM_2.5_ concentration, $AP_{it}$ denotes the mean PM_2.5_ concentration (μg/m^3^) in county during year, and denotes the average PM_2.5_ concentration in county during year. The basic data for this process are 2010 data extracted from the previous research, which are approximately 54,730 in the CONUS in 2010, 54,905 in the whole U.S.^2^. The previous EPA report indicates a 1 μg/m^3^ decrease in annual average PM_2.5_ concentration is associated with a from 0.7% to 1.6% decrease in annual all-cause mortality^45^. Another study claims that a 10-μg/m^3^ decrease in PM_2.5_ is related to a 6%—7% decrease in mortality among elderly individual each per year^46^. Since the basic data in 2010 is about premature deaths, we assume that the coefficient of the relationship between premature deaths and annual PM_2.5_ concentration, in Eq. (12), is 1.2%/(μg/m^3^), the roughly average value of 0.7%/(μg/m^3^) and 1.6%/(μg/m^3^). The percentage of the premature deaths due to PM_2.5_ from on-road transportation is estimated as follows:

13 $$PTA_{t} = MAPOR_{t} /MAP_{t} \times 100\%$$

where is the percentage of premature deaths due to PM_2.5_ from on-road transportation, is the total number of premature deaths due to PM_2.5_ from on-road transportation in the CONUS during year, and is the total number of premature deaths in the CONUS during year.

## Supplementary Information

Supplementary Information.

## Data Availability

All data sources used in the analyses, along with fully reproducible code, are publicly available at https://github.com/MichaelChaoLi-cpu/On-road_Transportation_PM2.5.git.
