# Supplementary material for: Contribution of on-road transportation to PM2.5
Source: Sci Rep. 2021 Oct 29;11:21320. doi: 10.1038/s41598-021-00862-x (PMC8556279; doi:10.1038/s41598-021-00862-x)
Supplement: Supplementary file 1 — Supplementary Information. [file 41598_2021_862_MOESM1_ESM.pdf]

Supplementary Materials for

Contribution of On-Road Transportation to $PM_{2.5}$

Authors

Chao Li[1], Shunsuke Managi*[1]

Affiliations

[1] Urban Institute & School of Engineering, Kyushu University, Japan

* Correspondent to: Shunsuke Managi, managi@doc.kyushu-u.ac.jp, Kyushu University

744 Motooka, Nishi-ku, Fukuoka 819-0395 Japan

Table S1: Data Statistic Summary

Statistic	N	Mean	St Dev	Min	Max
**$PM_{2.5}$ (μg/m^3)**	49,712	9.611	2.393	2.705	21.742
CO_2 Emission on Road(Million Tons/km^2)	49,712	0.408	1.099	0.0004	19.312
Open Space Developed Area (%)	49,712	4.319	3.697	0.135	34.904
Low Intensity Developed Area (%)	49,712	2.392	4.072	0.014	42.847
Medium Intensity Developed Area (%)	49,712	1.079	2.831	0.001	33.733
High Intensity Developed Area (%)	49,712	0.468	1.887	0.000	43.104
Open Water (%)	49,712	4.845	11.507	0.000	90.995
Woody Wetlands (%)	49,712	5.428	9.012	0.000	68.865
Emergent Herbaceous Wetlands (%)	49,712	1.370	3.449	0.000	60.514
Deciduous Forest (%)	49,712	15.988	19.444	0.000	88.061
Evergreen Forest (%)	49,712	8.708	13.758	0.000	83.679
Mixed Forest (%)	49,712	5.301	7.245	0.000	57.404
Shrub (%)	49,712	8.420	18.232	0.000	98.928
Grassland (%)	49,712	9.438	17.136	0.0005	97.739
Pasture (%)	49,712	10.404	12.787	0.000	82.365
Cultivated Crops (%)	49,712	21.442	26.266	0.000	92.842
Population Density (Thousand Capita/km^2)	49,712	0.065	0.406	0.00003	16.043
Mean Of Daily Temperature In Summer (K degree)	49,712	303.153	3.499	289.283	314.638
Mean Of Daily Temperature In Winter (K degree)	49,712	280.291	6.864	260.412	299.867
Mean Of Relative Humidity In Summer (%)	49,712	88.789	10.791	26.484	100.000
Mean Of Relative Humidity In Winter (%)	49,712	87.392	5.887	40.111	99.855
$PM_{2.5}$-Related Mortality in 2010	3,107	17.615	90.771	0.000	3611.315

Table S2: Data Sources

Variable	Data Source	Data Type	Period
$PM_{2.5}$ (μg/m^3)	the Centers for Disease Control and Prevention (CDC): https://catalog.data.gov/dataset/daily-pm2-5-concentrations-all-county-2001-2016-ee6c2	County-level daily concentration	2001 - 2016
CO2 Emission on Road(Tons)	National Aeronautics and Space Administration (NASA): https://daac.ornl.gov/cgi-bin/dsviewer.pl?ds_id=1735	1-km raster	2001 - 2016
Open Space Developed Area<br>Low Intensity Developed Area<br>Medium Intensity Developed Area<br>High Intensity Developed Area<br>Open Water<br>Woody Wetlands<br>Emergent Herbaceous Wetlands<br>Deciduous Forest<br>Evergreen Forest<br>Mixed Forest<br>Shrub<br>Grassland<br>Pasture<br>Cultivated Crops	National Land Cover Dataset (NLCD): https://www.mrlc.gov/data/nlcd-land-cover-conus-all-years	30-m raster	2001, 2004, 2006, 2008, 2011, 2013, 2016
Population Density (Thousand Capita)	WorldPop: https://www.worldpop.org/project/categories?id=18	1-km raster	2001 - 2016
Mean Of Daily Temperature In Summer (K °)<br>Mean Of Daily Temperature In Winter (K °)<br>Mean Of Relative Humidity In Summer (%)<br>Mean Of Relative Humidity In Winter (%)	Gridmet via Google Earth engine: http://www.climatologylab.org/gridmet.html	4-km raster	2001 - 2016
$PM_{2.5}$-Related Mortality in 2010	Research from Nature	5-km raster	2010
County Boundaries	the U.S. Census Bureau: https://www2.census.gov/geo/tiger/GENZ2016/shp/	Shape file	2016

Note: Except $PM_{2.5}$ and air-pollution-related mortality in 2010, other variables are geographically average data.

Table S3: The Results of FEM and SPEM

Dependent Variable	$PM_{2.5}$ FEM	$PM_{2.5}$ SPEM
CO_2 Emission on Road(Million Tons/km^2)	0.657^{***}	0.534^{***}
	(0.035)	(0.018)
Open Space Developed Area (%)	1.198^{***}	-0.032^{***}
	(0.117)	(0.007)
Low Intensity Developed Area (%)	0.639^{***}	0.016^{**}
	(0.164)	(0.008)
Medium Intensity Developed Area (%)	1.864^{***}	-0.215^{***}
	(0.138)	(0.011)
High Intensity Developed Area (%)	-5.605^{***}	-0.026^{*}
	(0.219)	(0.015)
Open Water (%)	0.450^{***}	-0.069^{***}
	(0.053)	(0.006)
Woody Wetlands (%)	-0.059	-0.077^{***}
	(0.071)	(0.006)
Emergent Herbaceous Wetlands (%)	0.475^{***}	-0.154^{***}
	(0.070)	(0.006)
Deciduous Forest (%)	1.965^{***}	-0.039^{***}
	(0.084)	(0.006)
Evergreen Forest (%)	1.210^{***}	-0.058^{***}
	(0.083)	(0.006)
Mixed Forest (%)	0.281^{***}	-0.070^{***}
	(0.090)	(0.006)
Shrub (%)	1.143^{***}	-0.094^{***}
	(0.083)	(0.006)
Grassland (%)	1.198^{***}	-0.099^{***}
	(0.082)	(0.006)
Pasture (%)	1.826^{***}	-0.057^{***}
	(0.082)	(0.006)
Cultivated Crops (%)	1.016^{***}	-0.051^{***}
	(0.083)	(0.006)
Population Density (Thousand Capita/km2)	1.451^{**}	-0.319^{***}
	(0.669)	(0.045)
Mean Of Daily Temperature In Summer	0.031^{***}	0.114^{***}
	(0.004)	(0.004)
Mean Of Daily Temperature In Winter	-0.070^{***}	0.022^{***}
	(0.003)	(0.002)
Mean Of Relative Humidity In Summer	-0.003^{**}	0.033^{***}
	(0.001)	(0.001)
Mean Of Relative Humidity In Winter	0.017^{***}	0.009^{***}
	(0.002)	(0.002)
ρ		0.114***

		(0.007)
Observations	49,712	49,712
R^2	0.366	0.491

* p<0.1, **p<0.5, ***p<0.01

Table S4: The Result of SPAM

Dependent variable:	$PM_{2.5}$		
	Direct Impact	Indirect Impact	Total Impact
CO2 Emission on Road(Million Tons/km2)	0.498***	0.097***	0.595***
	(0.017)	(0.005)	(0.020)
Open Space Developed Area (%)	-0.034***	-0.007***	-0.041***
	(0.007)	(0.001)	(0.008)
Low Intensity Developed Area (%)	0.014*	0.003*	0.017*
	(0.008)	(0.002)	(0.010)
Medium Intensity Developed Area (%)	-0.213***	-0.042***	-0.254***
	(0.011)	(0.003)	(0.014)
High Intensity Developed Area (%)	-0.014	-0.003	-0.017
	(0.014)	(0.003)	(0.017)
Open Water (%)	-0.066***	-0.013***	-0.079***
	(0.006)	(0.001)	(0.007)
Woody Wetlands (%)	-0.074***	-0.015***	-0.089***
	(0.006)	(0.001)	(0.007)
Emergent Herbaceous Wetlands (%)	-0.150***	-0.029***	-0.179***
	(0.006)	(0.002)	(0.008)
Deciduous Forest (%)	-0.036***	-0.007***	-0.043***
	(0.006)	(0.001)	(0.007)
Evergreen Forest (%)	-0.055***	-0.011***	-0.065***
	(0.006)	(0.001)	(0.007)
Mixed Forest (%)	-0.068***	-0.013***	-0.081***
	(0.006)	(0.001)	(0.007)
Shrub (%)	-0.088***	-0.017***	-0.106***
	(0.006)	(0.001)	(0.007)
Grassland (%)	-0.094***	-0.018***	-0.112***
	(0.006)	(0.001)	(0.007)
Pasture (%)	-0.054***	-0.011***	-0.065***
	(0.006)	(0.001)	(0.007)
Cultivated Crops (%)	-0.047***	-0.009***	-0.056***
	(0.006)	(0.001)	(0.007)
Population Density (Thousand Capita/km2)	-0.303***	-0.059***	-0.363***
	(0.041)	(0.008)	(0.049)
Mean Of Daily Temperature In Summer	0.116***	0.023***	0.138***
	(0.004)	(0.001)	(0.005)
Mean Of Daily Temperature In Winter	0.017***	0.003***	0.021***
	(0.002)	(0.0005)	(0.003)
Mean Of Relative Humidity In Summer	0.034***	0.007***	0.041***
	(0.001)	(0.0003)	(0.001)
Mean Of Relative Humidity In Winter	0.010***	0.002***	0.012***
	(0.002)	(0.0003)	(0.002)
**Note:**			* p<0.1, **p<0.5, ***p<0.01

Table S5: The Result from SPDM

Dependent variable:		$PM_{2.5}$	
	Direct Impact	Indirect Impact	Total Impact
CO_2 Emission on Road(Million Tons/km^2)	0.459^{***}	0.188^{***}	0.646^{***}
	(0.018)	(0.010)	(0.027)
Open Space Developed Area (%)	-0.032^{***}	-0.013^{***}	-0.045^{***}
	(0.007)	(0.003)	(0.010)
Low Intensity Developed Area (%)	0.018^{**}	0.007^{**}	0.026^{**}
	(0.008)	(0.003)	(0.011)
Medium Intensity Developed Area (%)	-0.216^{***}	-0.088^{***}	-0.305^{***}
	(0.011)	(0.005)	(0.015)
High Intensity Developed Area (%)	-0.002	-0.001	-0.002
	(0.015)	(0.006)	(0.021)
Open Water (%)	-0.062^{***}	-0.025^{***}	-0.088^{***}
	(0.006)	(0.002)	(0.008)
Woody Wetlands (%)	-0.071^{***}	-0.029^{***}	-0.100^{***}
	(0.006)	(0.002)	(0.008)
Emergent Herbaceous Wetlands (%)	-0.142^{***}	-0.058^{***}	-0.201^{***}
	(0.006)	(0.003)	(0.009)
Deciduous Forest (%)	-0.033^{***}	-0.013^{***}	-0.046^{***}
	(0.006)	(0.002)	(0.008)
Evergreen Forest (%)	-0.051^{***}	-0.021^{***}	-0.072^{***}
	(0.006)	(0.002)	(0.008)
Mixed Forest (%)	-0.065^{***}	-0.026^{***}	-0.091^{***}
	(0.006)	(0.002)	(0.008)
Shrub (%)	-0.083^{***}	-0.034^{***}	-0.116^{***}
	(0.006)	(0.002)	(0.008)
Grassland (%)	-0.087^{***}	-0.036^{***}	-0.123^{***}
	(0.006)	(0.002)	(0.008)
Pasture (%)	-0.050^{***}	-0.020^{***}	-0.071^{***}
	(0.006)	(0.002)	(0.008)
Cultivated Crops (%)	-0.044^{***}	-0.018^{***}	-0.062^{***}
	(0.006)	(0.002)	(0.008)
Population Density (Thousand Capita/km2)	-0.284^{***}	-0.116^{***}	-0.400^{***}
	(0.046)	(0.019)	(0.064)
Mean Of Daily Temperature In Summer	0.111^{***}	0.045^{***}	0.157^{***}
	(0.004)	(0.002)	(0.006)
Mean Of Daily Temperature In Winter	0.015^{***}	0.006^{***}	0.021^{***}
	(0.002)	(0.001)	(0.003)
Mean Of Relative Humidity In Summer	0.034^{***}	0.014^{***}	0.048^{***}
	(0.001)	(0.001)	(0.002)
Mean Of Relative Humidity In Winter	0.010^{***}	0.004^{***}	0.014^{***}
	(0.002)	(0.001)	(0.003)

* p<0.1, **p<0.5, ***p<0.01

Table S6: Statistical Test Parameter of Models

Model		R^2
FEM	Global	0.366
SPAM	Global	0.503
SPEM	Global	0.491
SPDM	Global	0.502
GTWR	Local	0.808

Note: Based on statistical test parameters, the SPAM is better than SPDM, but according to the locally robust panel Lagrange Multiplier tests, the spatial correlations of error term exists significantly. Therefore, we select SPDM as the best global model.

Table S7: Statistical Tests for Models Selection

Test	Model	p-value	Result	Mean of result	Note
Lagrange FF Multiplier Tests for Panel Models	Pooling Model	<0.001	Significant effects	Fixed or random effects models are better than pooling model	
F Test for Individual and/or Time Effects	Pooling model and fixed-effects model	<0.001	Significant effects	Fixed effects model is better than pooling model	
Hausman Test for Panel Models	Random and fixed effects model	<0.001	One model is inconsistent	Fixed effects model is better than random effects model	Without considering spatial correlations, fixed effects model is preferred.
Locally Robust Lagrange Multiplier test for Spatial Error Dependence	Fixed Effects Model	<0.001	Spatial error dependence	The spatial error dependence exists, using fixed effects model.	
Locally Robust Lagrange Multiplier test for Spatial Lag Dependence	Fixed Effects Model	<0.001	Spatial lag dependence	The spatial lag dependence exists, using fixed effects model.	Because both spatial error and lag dependence exists, the SPDM is the most reasonable.
Hausman Test for Spatial Models	SPDMs based on random and fixed effects model	<0.001	One model is inconsistent	The SPDM based on fixed effects model is better	

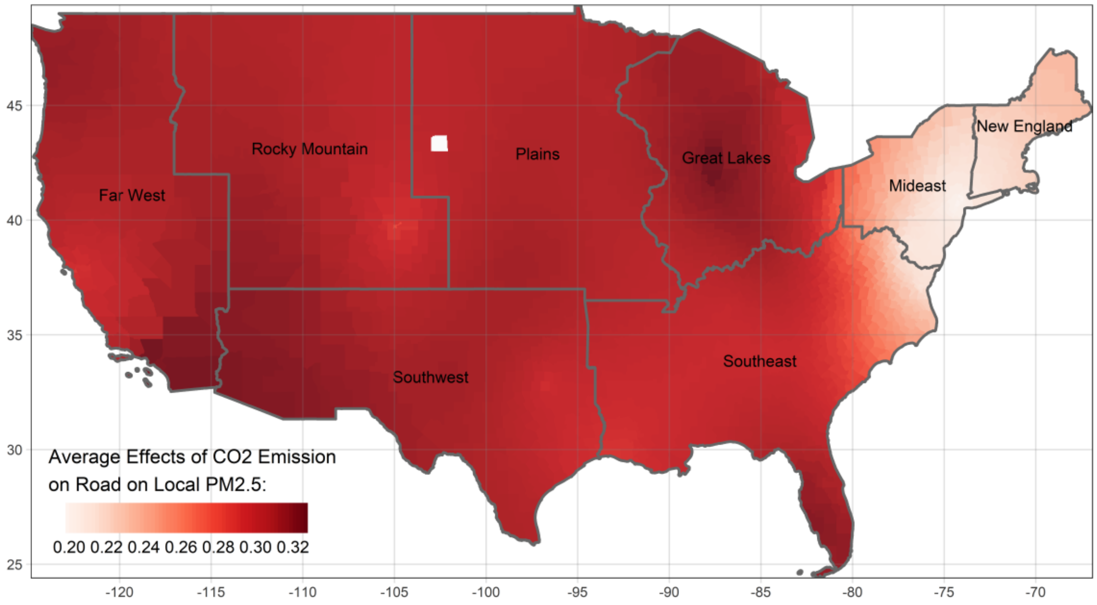

Figure S1: Average Impact of CO_2 Emission on Road on Local $PM_{2.5}$ (**Figure S1** is created by R 4.0.4, https://cran.r-project.org/bin/windows/base/old/4.0.4/)

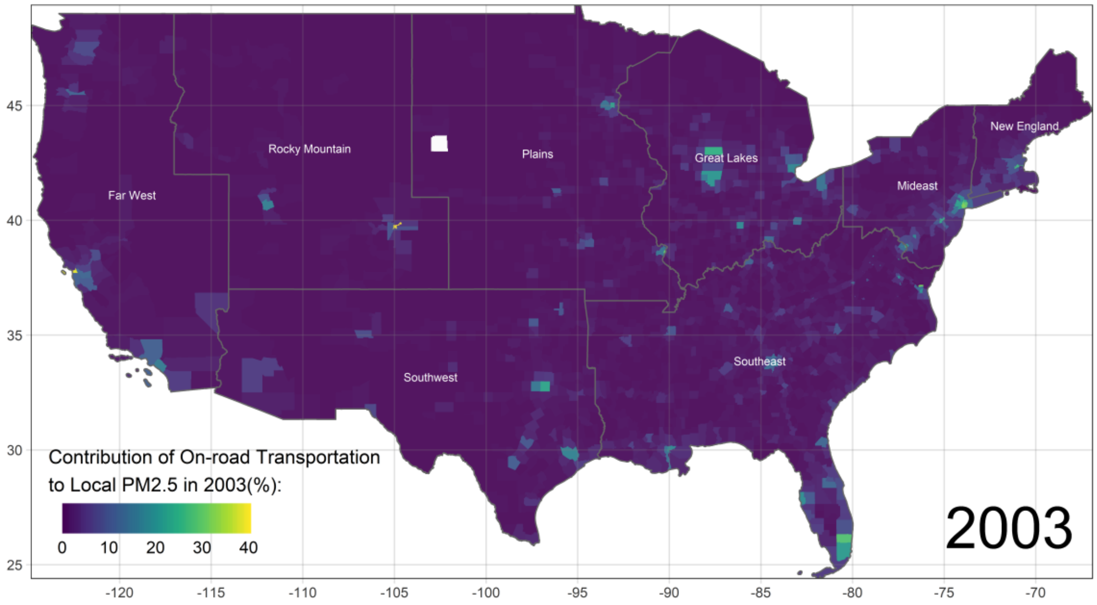

Figure S2.a: Contribution of On-road Transportation to County-level $PM_{2.5}$ (**Figure S2.a** is created by R 4.0.4, https://cran.r-project.org/bin/windows/base/old/4.0.4/)

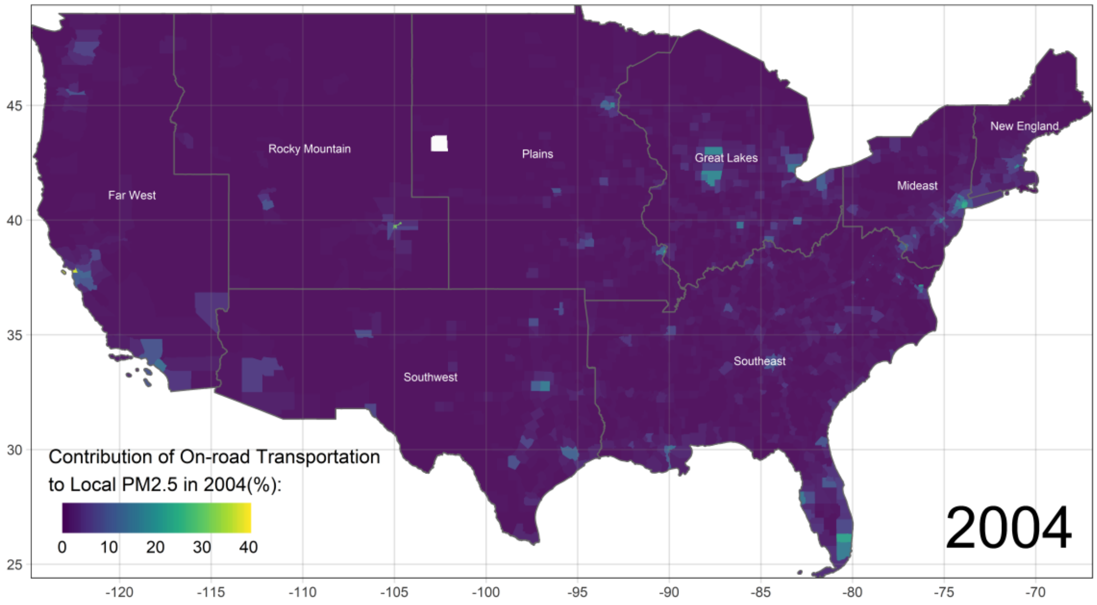

Figure S2.b: Contribution of On-road Transportation to County-level PM$_{2.5}$

(**Figure S2.b** is created by R 4.0.4, https://cran.r-project.org/bin/windows/base/old/4.0.4/)

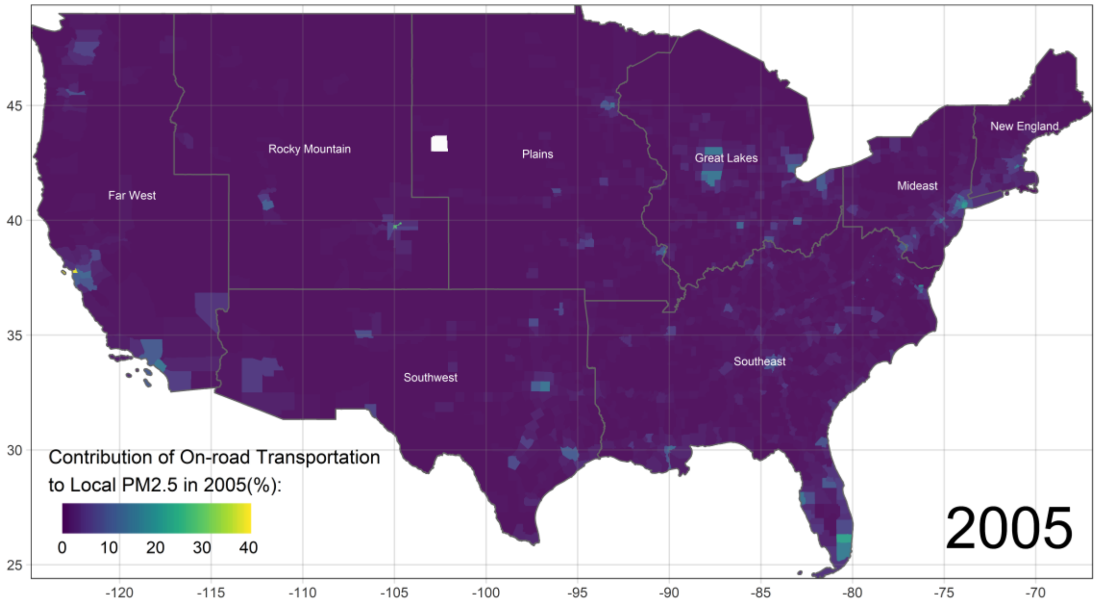

Figure S2.c: Contribution of On-road Transportation to County-level PM$_{2.5}$

(**Figure S2.c** is created by R 4.0.4, https://cran.r-project.org/bin/windows/base/old/4.0.4/)

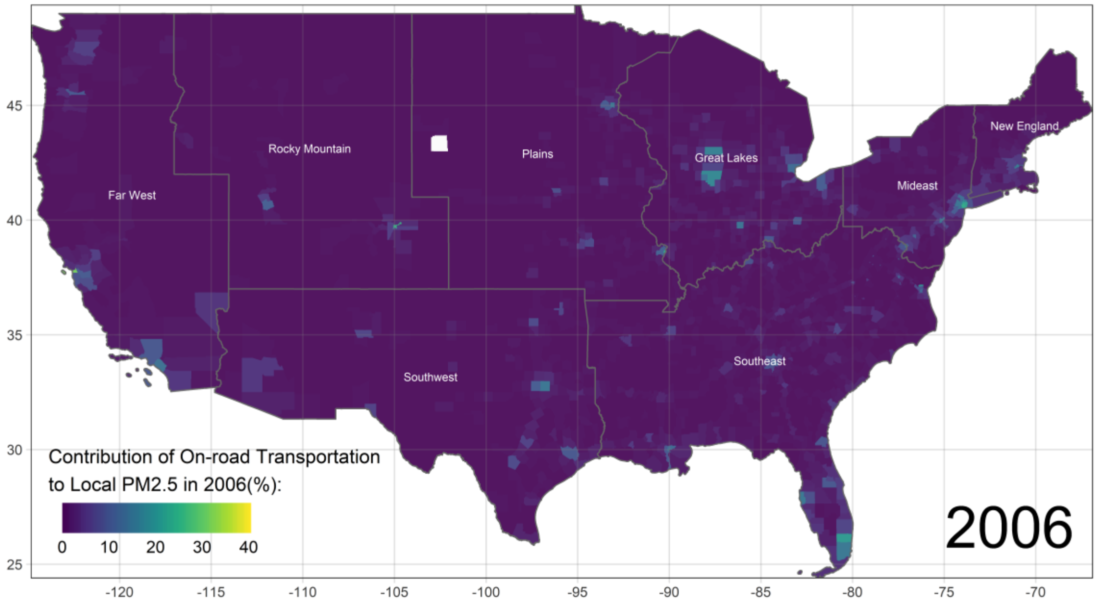

Figure S2.d: Contribution of On-road Transportation to County-level $PM_{2.5}$

(**Figure S2.d** is created by R 4.0.4, https://cran.r-project.org/bin/windows/base/old/4.0.4/)

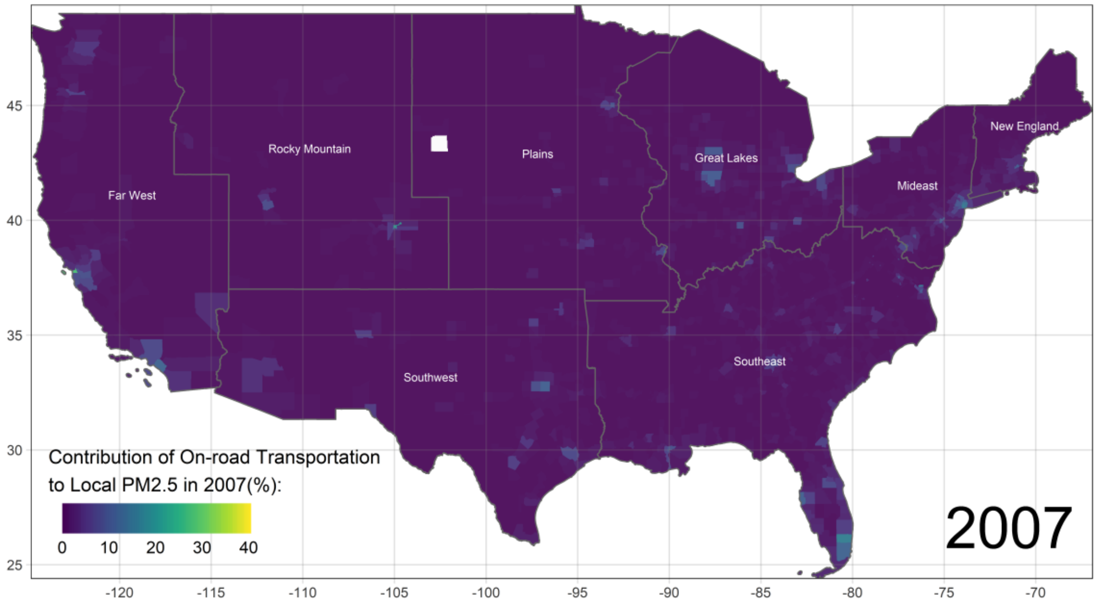

Figure S2.e: Contribution of On-road Transportation to County-level $PM_{2.5}$

(**Figure S2.e** is created by R 4.0.4, https://cran.r-project.org/bin/windows/base/old/4.0.4/)

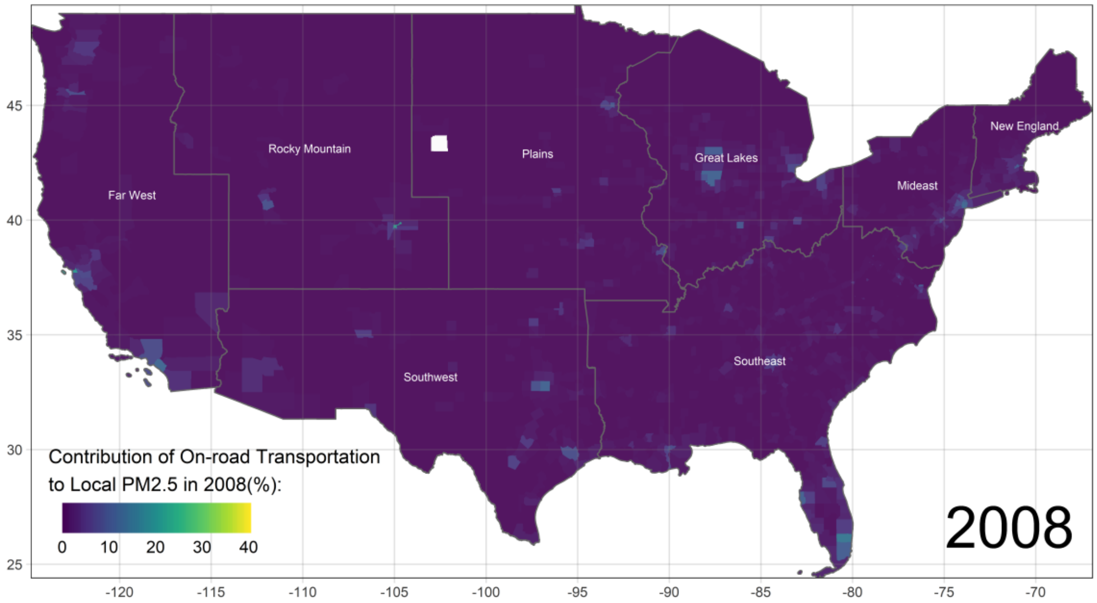

Figure S2.f: Contribution of On-road Transportation to County-level $PM_{2.5}$

(**Figure S2.f** is created by R 4.0.4, https://cran.r-project.org/bin/windows/base/old/4.0.4/)

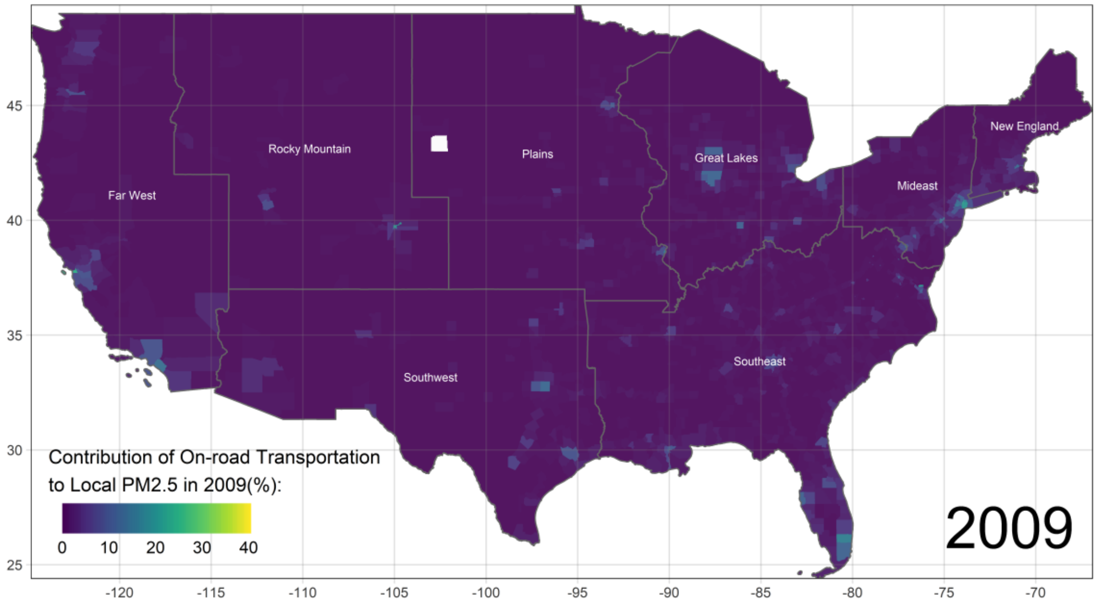

Figure S2.g: Contribution of On-road Transportation to County-level $PM_{2.5}$

(**Figure S2.g** is created by R 4.0.4, https://cran.r-project.org/bin/windows/base/old/4.0.4/)

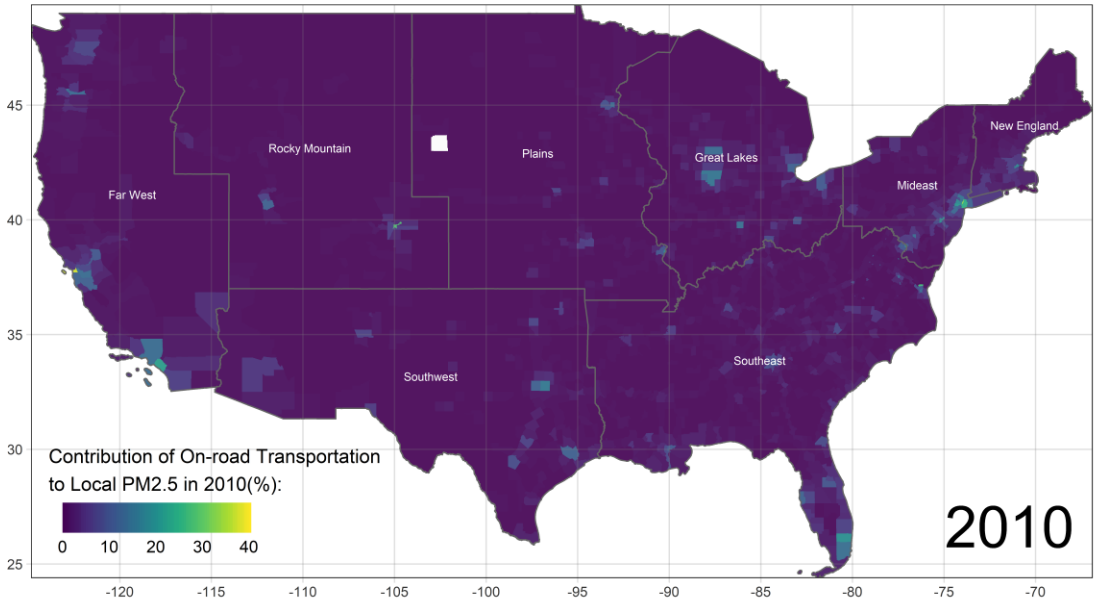

Figure S2.h: Contribution of On-road Transportation to County-level PM$_{2.5}$

(**Figure S2.h** is created by R 4.0.4, https://cran.r-project.org/bin/windows/base/old/4.0.4/)

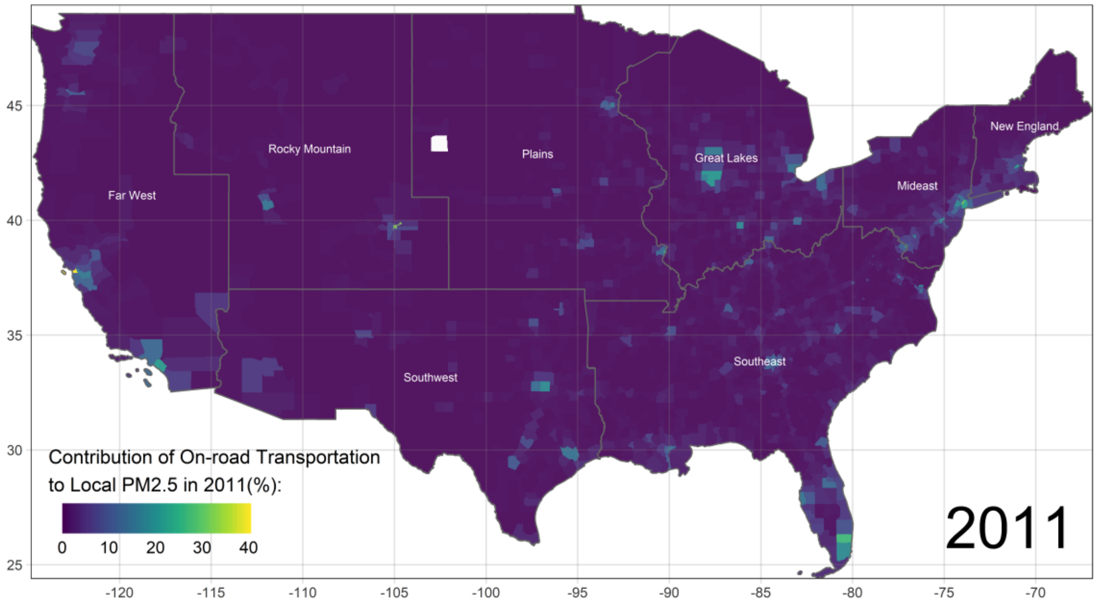

Figure S2.i: Contribution of On-road Transportation to County-level PM$_{2.5}$

(**Figure S2.i** is created by R 4.0.4, https://cran.r-project.org/bin/windows/base/old/4.0.4/)

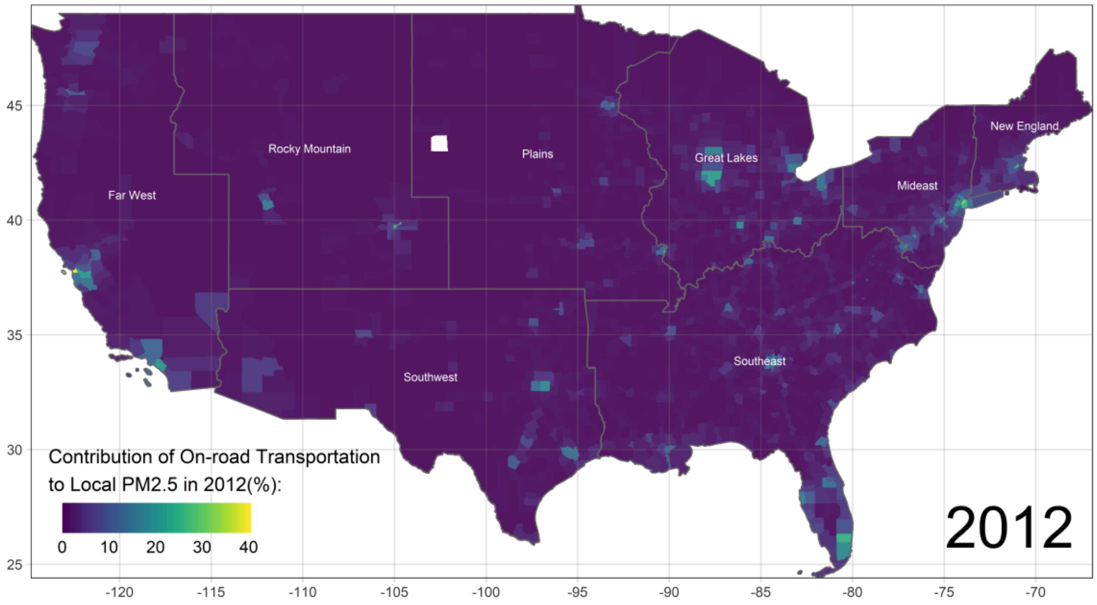

Figure S2.j: Contribution of On-road Transportation to County-level $PM_{2.5}$

(**Figure S2.j** is created by R 4.0.4, https://cran.r-project.org/bin/windows/base/old/4.0.4/)

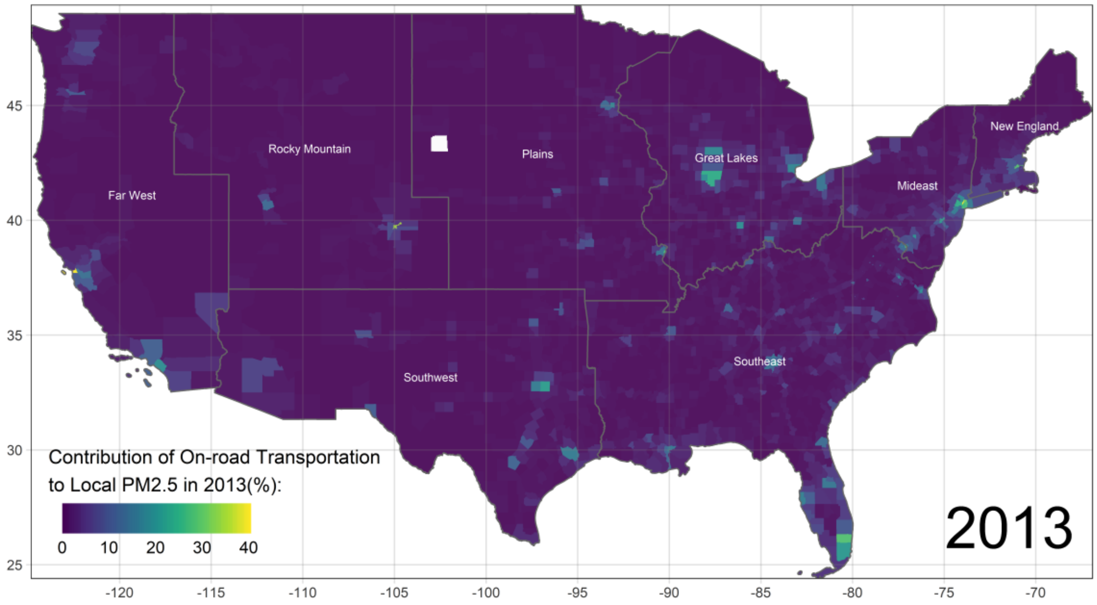

Figure S2.k: Contribution of On-road Transportation to County-level $PM_{2.5}$

(**Figure S2.k** is created by R 4.0.4, https://cran.r-project.org/bin/windows/base/old/4.0.4/)

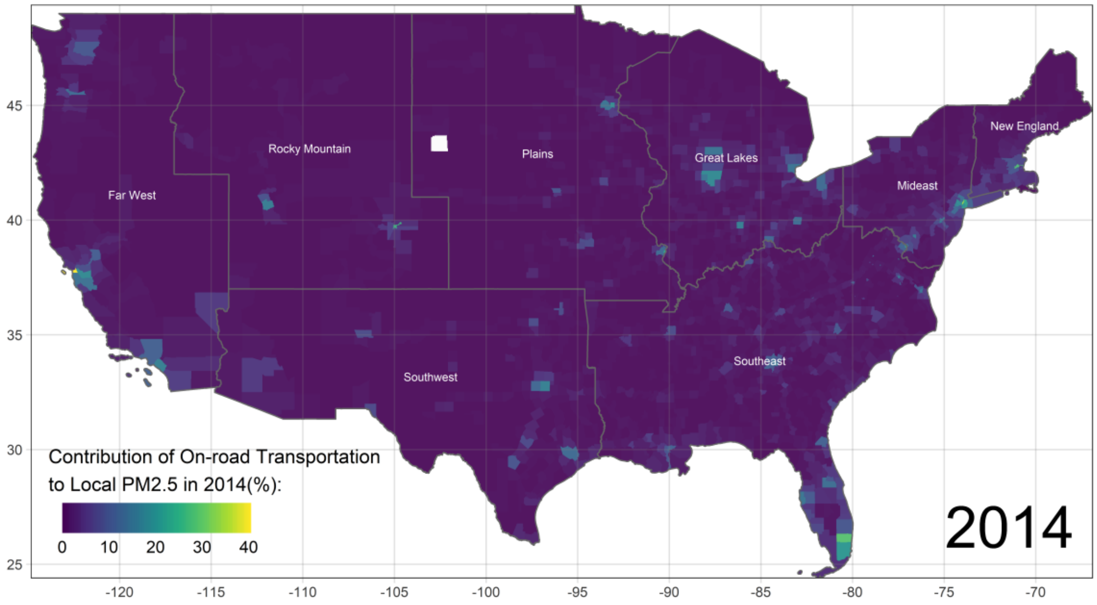

Figure S2.l: Contribution of On-road Transportation to County-level $PM_{2.5}$

(**Figure S2.l** is created by R 4.0.4, https://cran.r-project.org/bin/windows/base/old/4.0.4/)

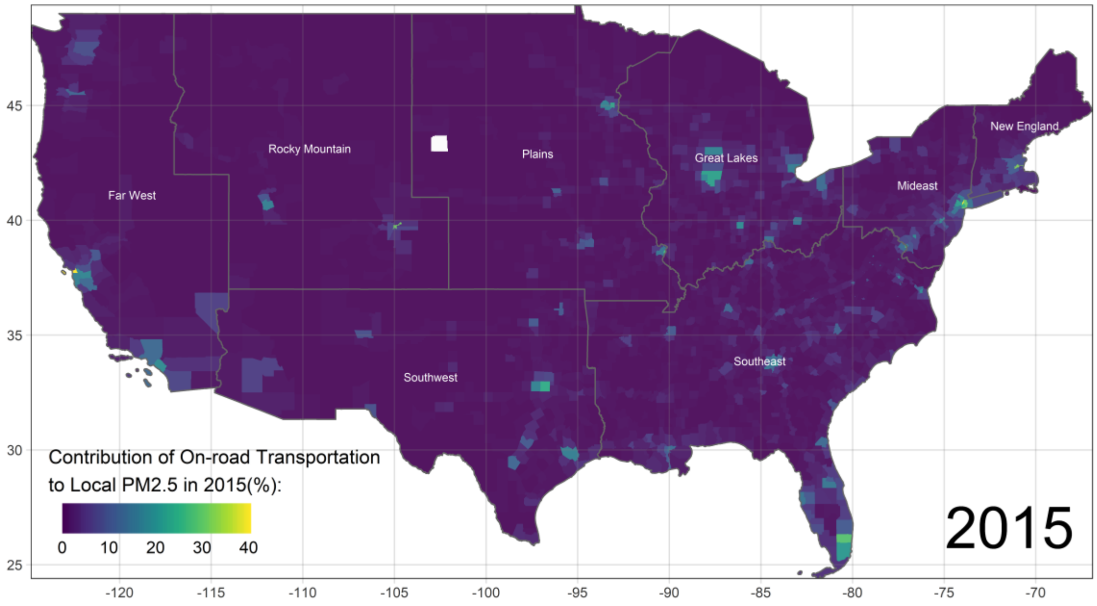

Figure S2.m: Contribution of On-road Transportation to County-level $PM_{2.5}$

(**Figure S2.m** is created by R 4.0.4, https://cran.r-project.org/bin/windows/base/old/4.0.4/)

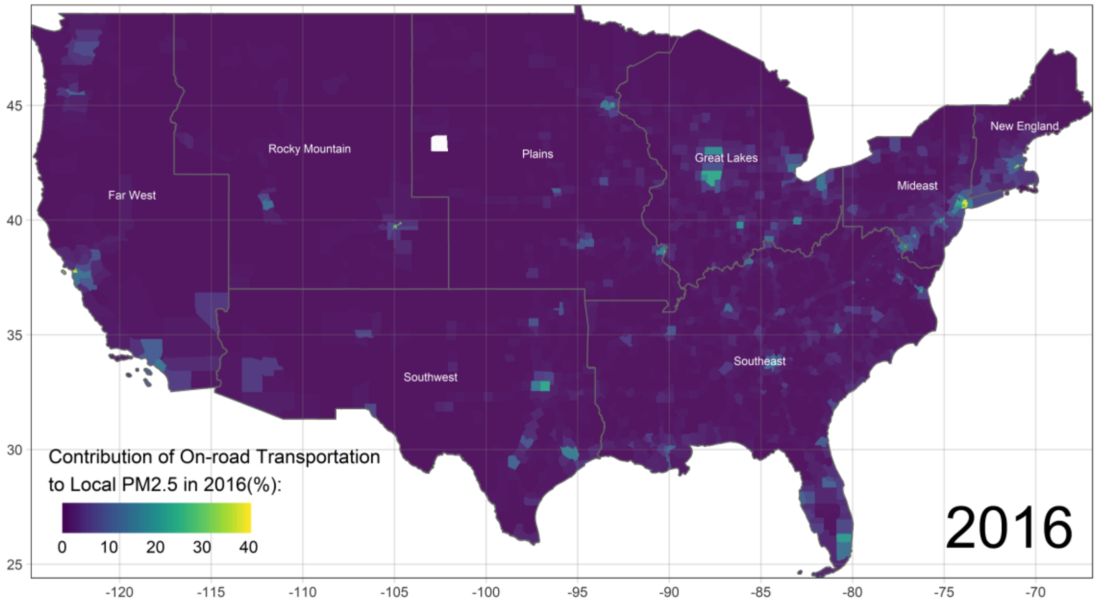

Figure S2.n: Contribution of On-road Transportation to County-level $PM_{2.5}$

(**Figure S2.n** is created by R 4.0.4, https://cran.r-project.org/bin/windows/base/old/4.0.4/)
